# Molecular Epidemiology of Feline and Human *Bartonella henselae* Isolates

**DOI:** 10.3201/eid1505.080995

**Published:** 2009-05

**Authors:** Rim Bouchouicha, Benoit Durand, Martine Monteil, B.B. Chomel, Moez Berrich, Mardjan Arvand, Richard J. Birtles, Edward B. Breitschwerdt, Jane E. Koehler, Ricardo Maggi, Soichi Maruyama, Rick Kasten, Elisabeth Petit, Henri-Jean Boulouis, Nadia Haddad

**Affiliations:** Ecole Nationale Vétérinaire d'Alfort, Maisons Alfort, France (R. Bouchouicha, M. Monteil, M. Berrich, E. Petit, H.-J. Boulouis, N. Haddad); Agence Française de Sécurité Sanitaire des Aliments, Maisons Alfort (B. Durand); University of California, Davis, California, USA (B. Chomel, R. Kasten); Zentrum für Gesundheitsschutz, Dillenburg, Germany (M. Arvand); University of Liverpool, Cheshire, UK (R.J. Birtles); North Carolina State University, Raleigh, North Carolina, USA (E. Breitschwerdt, R. Maggi); University of California, San Francisco, California, USA (J. Koehler); Nihon University, Kanagawa, Japan (S. Maruyama)

**Keywords:** Zoonoses, bacteria, Bartonella henselae, MLVA, VNTR, molecular epidemiology, cat-scratch disease, dispatch

## Abstract

Multiple locus variable number tandem repeat analysis was performed on 178 *Bartonella henselae* isolates from 9 countries; 99 profiles were distributed into 2 groups. Human isolates/strains were placed into the second group. Genotype I and II isolates shared no common profile. All genotype I isolates clustered within group B. The evolutive implications are discussed.

*Bartonella henselae* is the zoonotic agent of cat-scratch disease and has been associated with bacillary angiomatosis, bacillary peliosis, endocarditis, osteomyelitis, and neuroretinitis (*1*). It is usually present in low numbers in infected human tissues, whereas cats, the natural reservoir for the bacterium, are prone to persistent bacteremia. Therefore, most *B. henselae* isolates are of feline origin. Two genotypes, based on 16S rDNA differences, have been described (*1*). Genotype I was more frequently observed in humans in some countries where most cats are infected with genotype II (*2*,*3*). Genotype II is more prevalent in cats in Europe, Australia, and the United States (*2**–**6*), and most feline isolates from Asia belong to genotype I (*7**–**9*). In a recent study, 3 (1.6%) of 191 *B. henselae* isolates harbored 2 different 16S rDNA copies and could not be assigned to a distinct genotype (*10*). However, most isolates harbored 2 identical 16S rDNA copies and were assigned to either type I or II, confirming that delineation of *B. henselae* isolates in two 16S rDNA types is generally reasonable.

Multiple-locus variable number tandem repeat analysis (MLVA) was recently developed for *B. henselae* typing (*6*). The results are produced in an intrinsically quantitative form, called a profile, corresponding to the number of basic units in an isolate for each variable number tandem repeat (VNTR). MLVA was more discriminatory (*11*) than the other widely used typing techniques, such as pulsed-field gel electrophoresis (*2*), multilocus sequence typing (MLST) (*12*) and multispacer typing (MST) (*13*). In our protocol, MLVA involves the amplification of 5 main VNTR loci, BHV-A to -E, for *B. henselae* VNTRs (*6*).

## The Study

We analyzed 178 *B. henselae* isolates/strains from various sources (Table 1): 156 (88%) feline isolates/strains, 21 (11%) from diseased humans, and 1 isolate from a sick dog. The number of alleles varied from 7 (BHV-E) to 22 (BHV-B). Most of the European isolates (all but 1 of feline origin) (*2**,**4**,**6*) and of the American isolates/strains (North Carolina and California) (*5**,**14*), of which 85% were of feline origin, belonged to genotype II (89% and 64.6%, respectively). The Asian isolates (all but 1 of feline origin) (*7**–**9*) and the Australasian isolates (60% of human origin) (*12*), mainly belonged to genotype I (89.6% and 65%, respectively).

**Table 1:** Description of *Bartonella henselae* isolates and strains tested, global diversity of the typing system, and diversity variations according to 16S rDNA genotype, continent, and host*

Characteristics	No. isolates/ strains	No. profiles	Average no. isolates/profiles	DI	No. alleles (minimum–maximum no. repeats)				
					A	B	C	D	E
All *B. henselae*	178	99	1.8	0.98	8 (9–16)	22 (5–37)	14 (1–25)	8 (1–9)	7 (1–7)
16S rDNA genotype I	64	44	1.5	0.98	6	12	11	6	6
16S rDNA genotype II	114	55	2.0	0.97	6	15	7	7	6
Location									
Europe	80†	42	1.9	0.95	6	12	6	6	5
Asia	29‡	22	1.3	0.98	7	10	8	4	3
USA	49§	28	1.7	0.95	4	12	7	6	6
Australia–New Zealand	20¶	11	1.8	0.87	4	5	5	4	6
Host									
Human + dog	22#	12	1.8	0.87	2	6	6	3	6
Healthy cat	156	92	1.7	0.98	8	20	11	8	7

*DI, diversity index; A, BHV-A; B, BHV-B; C, BHV-C; D, BHV-D; E, BHV-E. †Denmark, 18 (*7*); France, 23 (*7*); Germany, 27 (*2*); UK, 12 (*5*). ‡Japan, 12 (*8*); Philippines, 7 (*9*); Thailand, 10 (*10*). §California: 36, including 5 owners and their 11 cats (*6*,*14*); North Carolina, 12 (provided by Ed Breitschwerdt). Reference strain Houston 1. ¶Australia, 18 and New Zealand, 2 (*3*,*12*). #21 human isolates from 1 German patient with bacillary angiomatosis (BA) (*2*), 1 Japanese patient with cat-scratch disease (provided by S. Maruyama), 12 Australian human patients with cat-scratch disease (*3*), 5 California human patients with BA (*14*), 1 North Carolina patient with a wide range of symptoms, including fatigue, joint pain, insomnia, headache, blurred vision, irritability (provided by Ed Breitschwerdt), plus the reference strain Houston 1 (ATCC 49882, initially isolated from a patient with BA) (*3*), and 1 isolate from a dog with endocarditis (provided by Ed Breitschwerdt).

Ninety-nine different MLVA profiles were observed (Table 1), corresponding to an average number of isolates per profile of 1.8 (Table 2). Sixty-nine of these profiles were found in only 1 isolate or strain (67%), and 30 were observed in >1 isolate. Among these, none was shared by genotype I and genotype II isolates. Diversity index (DI) was 0.98 (Table 1). Diversity was observed in both genotypes because genotype-specific DIs were almost identical (Table 1).

**Table 2:** Distribution of *Bartonella henselae* isolates/strains by 16S rDNA genotype, host, and location for profiles with >2 isolates*

VNTR profile					No. isolates	16S rDNA genotype			Host			Location			
A	B	C	D	E		I	II		Human	Healthy cat		Europe	Asia	USA	Aus-NZ
10	14	2	2	1	14		14			14		14			
9	15	2	1	1	8		8			8		5		3	
10	15	2	2	1	8		8			8		8			
14	34	2	7	4	8		8		1	7				8	
14	22	10	5	3	7	7			7						7
14	32	8	7	4	6		6		2	4				6	
14	20	10	7	5	4	4			2	2		3		1	
9	14	2	2	1	4		4			4		4			
10	15	2	1	1	4		4			4		4			
15	20	10	8	2	4	4				4			4		
13	14	6	5	4	3		3			3		3			
15	20	10	8	4	3	3			1	2				3	
13	31	6	5	5	2	2				2		2			
9	14	2	1	1	2		2			2		1		1	
9	15	2	2	1	2		2			2		2			
13	34	10	8	3	2	2				2			2		
14	36	8	7	4	2		2			2				2	
13	32	8	7	4	2		2			2				2	
9	15	2	1	3	2		2			2			1	1	
14	32	8	7	1	2		2		2						2
14	26	6	8	4	2		2			2					2
15	32	10	8	5	2	2				2					2
14	11	6	7	4	2		2			2		2			
13	20	7	8	2	2	2				2				2	
14	20	6	1	2	2	2			1	1				2	
10	15	3	1	1	2		2			2		2			
10	15	3	2	1	2		2			2		2			
14	20	10	8	2	2	2				2			2		
14	18	10	1	3	2	2				2			2		
15	20	10	1	2	2	2				2			2		

*VNTR, variable number tandem repeat; Aus-NZ, Australia and New Zealand; A, BHV-A; B, BHV-B; C, BHV-C; D, BHV-D; E, BHV-E.

MLVA profiles appeared location-specific because only 4 (13%) of the 30 profiles observed in >1 isolate/strain were present in >1 continent (Table 2). Within continents, no marked dominance of a given profile was observed, and continent-specific DIs were similar (Table 1).

Of the 99 *B. henselae* profiles, 12 were obtained from the 21 human isolates/strains and 1 from the dog, whereas 92 profiles were obtained from the 156 feline isolates. Five profiles were common to 5 human and 11 feline isolates. Among the 30 profiles observed in >2 isolates, 23 were observed only in feline isolates (Table 2). The proportion of genotype I profiles was significantly higher in human-specific profiles than in cat-specific profiles (p = 0.01, by Fisher test).

For BHV-A, only 2 alleles (14 and 15 copies) were found in isolates from humans, whereas all 8 identified alleles were observed in cat isolates. The number of repeats differed significantly between sick humans and healthy cats (p = 0.02, by Fisher test).

Relationships between the 99 MLVA profiles were analyzed by unweighted pair group method with arithmatic mean (UPGMA), using a categorical distance, with a *B. koehlerae* isolate used as an outgroup. To take into account that UPGMA is sensitive to taxa entry order, we computed the majority-rule consensus tree of 500 dendrograms built with random taxa entry order. MLVA profiles were grouped into 2 main groups named A and B (Appendix Figure). Group A (26 profiles), was exclusively constituted by genotype II feline isolates. Group B (73 profiles), to which all human isolates belonged, further divided in 2 subgroups, Ba and Bb. Subgroup Ba (38 profiles) was exclusively composed of genotype I isolates, including the reference strain Houston I and a homogenous subgroup, Ba1, containing 84% of the Asian isolates. Finally, 83% of subgroup Bb isolates belonged to genotype II (29/35 profiles).

The utility of MLVA for molecular epidemiologic analysis of clusters was tested using isolates from California cats and their owners (*14*). Five human–cat groups of *B. henselae* isolates were analyzed. For 1 cat-human pair of isolates, which belonged, respectively, to genotype II and genotype I, major profile differences were observed, as expected. The 4 other cat-human groups, which possessed the same genotype, also had the same MLVA profile with the 5 tested BHV, as well as with the 6 additional BHV (F–K) and variant alleles for BHV-A and/or B (*6*). Sequencing confirmed these results.

## Conclusions

Our results confirm that VNTRs are excellent molecular markers for confirming or excluding the responsibility of a given cat in the transmission of *B. henselae* to a human. In California, the profile identity observed within 4 clusters further supports the hypothesis that all these humans acquired infection from their respective domestic cat contacts.

MLVA enabled a clear separation between genotypes I and II, because no profile was shared between both genotypes. The dendrogram showed a high level of discrimination between 16S rDNA genotypes in the *B. henselae* population tested. Interestingly, the groups and subgroups delineated by MLVA were the same as those defined by MLST, a standard method for phylogenetic analysis (*12*). The same was observed with MST (*13*). The isolates of the subgroup Bb appeared divergent and distant from each other and from subgroup Ba that contains almost all genotype I profiles (98%). Moreover and despite possible clustering for some of the isolates, none of the 21 human isolates was present in group A. Interestingly, as for most of the human patients, the isolate obtained from the ill dog also belonged to genotype I.

These observations suggest that all genotype I isolates could be phylogenetically derived from genotype II isolates located in group B but not in group A, as already suggested using MLST (*15*). This observation could mean that genotype II isolates belonging to group B are closer to genotype I isolates than to genotype II isolates belonging to group A; it also raises an important clinical question: Are feline genotype II isolates belonging to group A nonpathogenic for humans? Genotype I isolates could represent the most pathogenic isolates for humans within a group of potentially zoonotic isolates, all belonging to group B and could represent an ultimate evolutionary step toward human infection. Additionally, within group B, the differences in the number of BHV-A repeat units observed between isolates from patients (humans, dog) versus cat isolates suggest that this specific VNTR could constitute a marker for the ability to cross the species barrier from reservoir cats to susceptible species, independent of the 16S rDNA genotype.

## Supplementary Material

Appendix Figure Dendrogram of the 99 multilocus variable number tandem repeat analysis (MLVA) profiles obtained with 178 isolates and strains. Bootstrap distances are indicated between brackets after the names of the groups and subgroups. The numbers in the brackets after the number of isolates for each profile correspond to the number of isolates from humans and from cats, respectively. As, Asia; Au, Australia-New Zealand; Eu, Europe; US, United States; A, group A; B, group B; Ba, subgroup Ba; Bb, subgroup Bb. *Dog isolate.
