# Supplementary figures and images for: Molecular Epidemiology of Feline and Human Bartonella henselae Isolates
Source: Emerg Infect Dis. 2009 May;15(5):813–6. doi: 10.3201/eid1505.080995 (PMC2687025; doi:10.3201/eid1505.080995)

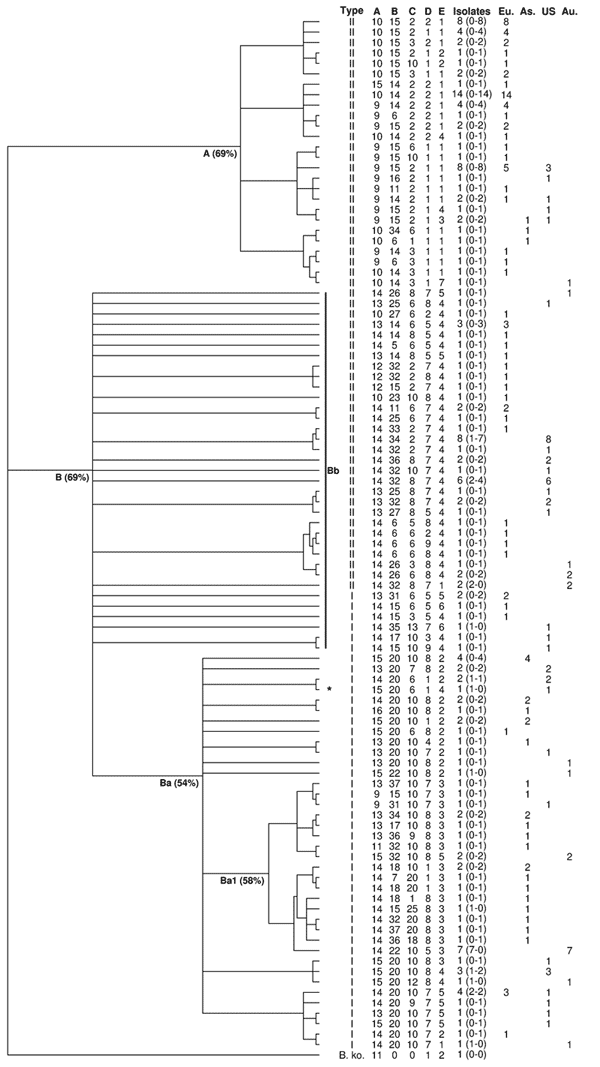

Supplement: Appendix Figure — Dendrogram of the 99 multilocus variable number tandem repeat analysis (MLVA) profiles obtained with 178 isolates and strains. Bootstrap distances are indicated between brackets after the names of the groups and subgroups. The numbers in the brackets after the number of isolates for each profile correspond to the number of isolates from humans and from cats, respectively. As, Asia; Au, Australia-New Zealand; Eu, Europe; US, United States; A, group A; B, group B; Ba, subgroup Ba; Bb, subgroup Bb. *Dog isolate. [file 08-0995_app-s1.gif]
